# Effect of salinity on scytonemin yield in endolithic cyanobacteria from the Atacama Desert

**DOI:** 10.1038/s41598-024-60499-4

**Published:** 2024-04-28

**Authors:** María Cristina Casero, María Ángeles Herrero, Juan Pablo De la Roche, Antonio Quesada, David Velázquez, Samuel Cirés

**Affiliations:** 1 Departamento de Biología, Universidad Autónoma de Madrid, 28014 Madrid, Spain; 2 Microalgae Solutions S.L., Calle Dehesa vieja, 8, N5, 28052 Madrid, Spain

**Keywords:** Applied microbiology, Biotechnology, Microbial ecology

## Abstract

Cyanobacteria inhabiting extreme environments constitute a promising source for natural products with biotechnological applications. However, they have not been studied in-depth for this purpose due to the difficulties in their isolation and mass culturing. The Atacama Desert suffers one of the highest solar irradiances that limits the presence of life on its hyperarid core to endolithic microbial communities supported by cyanobacteria as primary producers. Some of these cyanobacteria are known to produce scytonemin, a UV-screening liposoluble pigment with varied biotechnological applications in cosmetics and other industries. In this work we carried out a strain selection based on growth performance among 8 endolithic cyanobacteria of the genera *Chroococcidiopsis*, *Gloeocapsa* and *Gloeocapsopsis* isolated from non-saline rocks of the Atacama Desert. Then we investigated the influence of NaCl exposure on scytonemin production yield. Results in the selected strain (*Chroococcidiopsis* sp. UAM571) showed that rising concentrations of NaCl lead to a growth decrease while triggering a remarkable increase in the scytonemin content, reaching maximum values at 20 g L^−1^ of NaCl over 50-fold higher scytonemin contents than those obtained without NaCl. Altogether, these findings point out to cyanobacteria from the Atacama Desert as potentially suitable candidates for pilot-scale cultivation with biotechnological purposes, particularly to obtain scytonemin.

## Introduction

Microorganisms inhabiting extreme environments, both extremophile and extremotolerant strains, have become a promising source of new bioactive compounds of interest^1^. The capacity of producing these interesting products is explained by their survival strategies to deal with harsh environmental conditions and to achieve competitive advantages in those extreme environments where essential resources are also scarce or difficult to uptake^2^. Thus, the genetic adaptation of microorganisms inhabiting extreme conditions could be expected to allow the synthesis of novel metabolites with unique structures and specific biological activity^3^, helping in their colonization^4^.

Among environmental factors acting in extreme habitats, it is well known that UVR exerts lethal effects on biological systems since it is absorbed by biomolecules^5^. Thus, some cyanobacteria are able to produce scytonemin, the most widespread sunscreen pigment exclusively produced by these organisms^6,7^. It is a yellow–brown lipid-soluble dimeric compound composed of indolic and phenolic subunits^8^ that occurs in both oxidized (MW 544 Da) and reduced (MW 546 Da) forms which in vivo absorption maximum is at 370 nm and purified at 386 nm, in the UVA region. This pigment is located in the exopolysaccharidic (EPS) sheath of certain terrestrial cyanobacterial species and is highly stable under different abiotic stresses being able to reduce about 90% of the radiation that reaches the inside of cells^9^. Scytonemin production is not constitutive, occurring in response to adverse environmental conditions, such as UV radiation, oxidative stress^10^, nutrient deficits^11^, and desiccation and salt stress^10–12^, whose physiological effects and responses are considered to be related^13^. Its biosynthetic pathway operon has been poorly studied, first described in 2007 by Soule et al.^14^ in *Nostoc punctiforme* ATCC 29133 and subsequently in genera *Anabaena*, *Rivularia*, *Scytonema*, *Calothrix*, *Nodularia*, *Chlorogloeopsis* and *Lyngbya*, the cluster comprises 18 genes, including genes coding for scytonemin precursors, scytonemin assembly (*scyA*, *scyB*, *scyC*, *scyD*, *scyE* and *scyF*) and regulators. This cluster presents a mosaic structure, differing in gene content, organization, and direction among species^15^. Among those genes, *scyC* gene has been the most studied and used for phylogenetic studies^16^. *ScyC* is essential for scytonemin formationcatalysing the cyclization and decarboxylation of a β-ketoacid to form a ketone in the early stages of scytonemin biosynthesisfor its^17^. Furthermore, a prospective study using *Nostoc punctiforme* genes pointed out to *scyC* along with *scyA* and *scyB* as the only three genes required for heterologous production of scytonemin in *E. coli*^18^.

Scytonemin has become of particular interest to the cosmetic industry due to its UV-absorbing and antioxidant capacity. Moreover, its hydrophobic nature could facilitate penetration into the skin, thus increasing protection against harmful sun radiation^18^. It also has antitumor potential, being capable of inducing apoptosis in tumor cells by inhibiting the PLK1 (polo-like kinase 1) enzyme, which is involved in the development of several types of cancer, such as osteosarcoma and myeloma^19,20^.

The presence of cyanobacteria able to produce scytonemin has been reported in the endolithic microbial communities of some deserts^21,22^. Within those, the Atacama Desert (North Chile) is perhaps the most challenging polyextreme environment on Earth holding the highest surface ultraviolet radiation (UVR), photosynthetic active radiation (PAR) and annual mean surface solar radiation^23^. Under these extreme conditions in this desert, life is often limited to endolithic microbial communities (inside the rocks) supported by primary producers, mainly cyanobacteria, some of which are highly resistant to desiccation, light overexposure, and ionizing radiation^24^. However, the capacity of cyanobacterial strains from Atacama to produce this UV-screening compound when exposed to increasing NaCl concentrations remains unexplored. Besides its role in osmotic stress, NaCl addition to the culture medium is often used to reduce microbial contamination in open cultures for biotechnological purposes.

Taking all these aspects into account, this work addresses (i) a cyanobacterial strain selection among 8 endolithic strains of the genera *Chroococcidiopsis*, *Gloeocapsa* and *Gloeocapsopsis,* which have been isolated from the Atacama Desert, characterizing their growth rate and scytonemin synthesis capacity, along with (ii) the effect of NaCl exposure in scytonemin synthesis.

## Results

### Strain selection

Growth rate varied between endolithic cyanobacterial strains (Table 1, Fig. S1). Thus, *Chroococcidiopsis* sp. UAM570 and UAM571 together with *Gloeocapsa* sp. UAM572 reached the highest growth rate (0.24 day^−1^), while the lowest was measured for *Chroococcidiopsis* sp. UAM580 (0.17 day^−1^). The only strain belonging to the genus *Gloeocapsopsis,* UAM573, showed an average growth rate (0.22 day^−1^).

**Table 1:** Growth rate (day^−1^) of 8 endolithic cyanobacterial strains of the Atacama Desert growth in BG11 culture medium.

Strain	Code	Growth rate (day^−1^)
*Chroococcidiopsis* sp.	UAM569	0.23 ± 0.01
*Chroococcidiopsis* sp.	UAM570	0.24 ± 0.01
*Chroococcidiopsis* sp.	UAM571	0.24 ± 0.00
*Gloeocapsa* sp.	UAM572	0.24 ± 0.00
*Gloeocapsopsis* sp.	UAM573	0.22 ± 0.00
*Chroococcidiopsis* sp.	UAM579	0.21 ± 0.00
*Chroococcidiopsis* sp.	UAM580	0.17 ± 0.01
*Chroococcidiopsis* sp.	UAM586	0.22 ± 0.00

Values are expressed as mean ± standard deviation (SD) (n = 3).

Since there was more than one strain that exhibited the highest growth rate, growth experiments exposing *Chroococcidiopsis* sp. UAM571 and *Gloeocapsa* sp. UAM572 to NaCl were performed (Table S1, Fig. S2). *Chroococcidiopsis* sp. UAM571 exhibited a higher growth rate for all NaCl concentrations and was then selected to perform the scytonemin production experiment.

### Scytonemin production by *Chroococcidiopsis* sp. UAM571 exposed to NaCl

To broadly understand the scytonemin production of *Chroococcidiopsis* sp. UAM571 three different features were studied: (i) content per biomass DW (mg scytonemin g DW^−1^), (ii) concentration per volume of culture (g scytonemin·L^−1^), and (iii) productivity, which means related to culture volume and time (µg scytonemin L^−1^ day^−1^).

**Figure 1:**
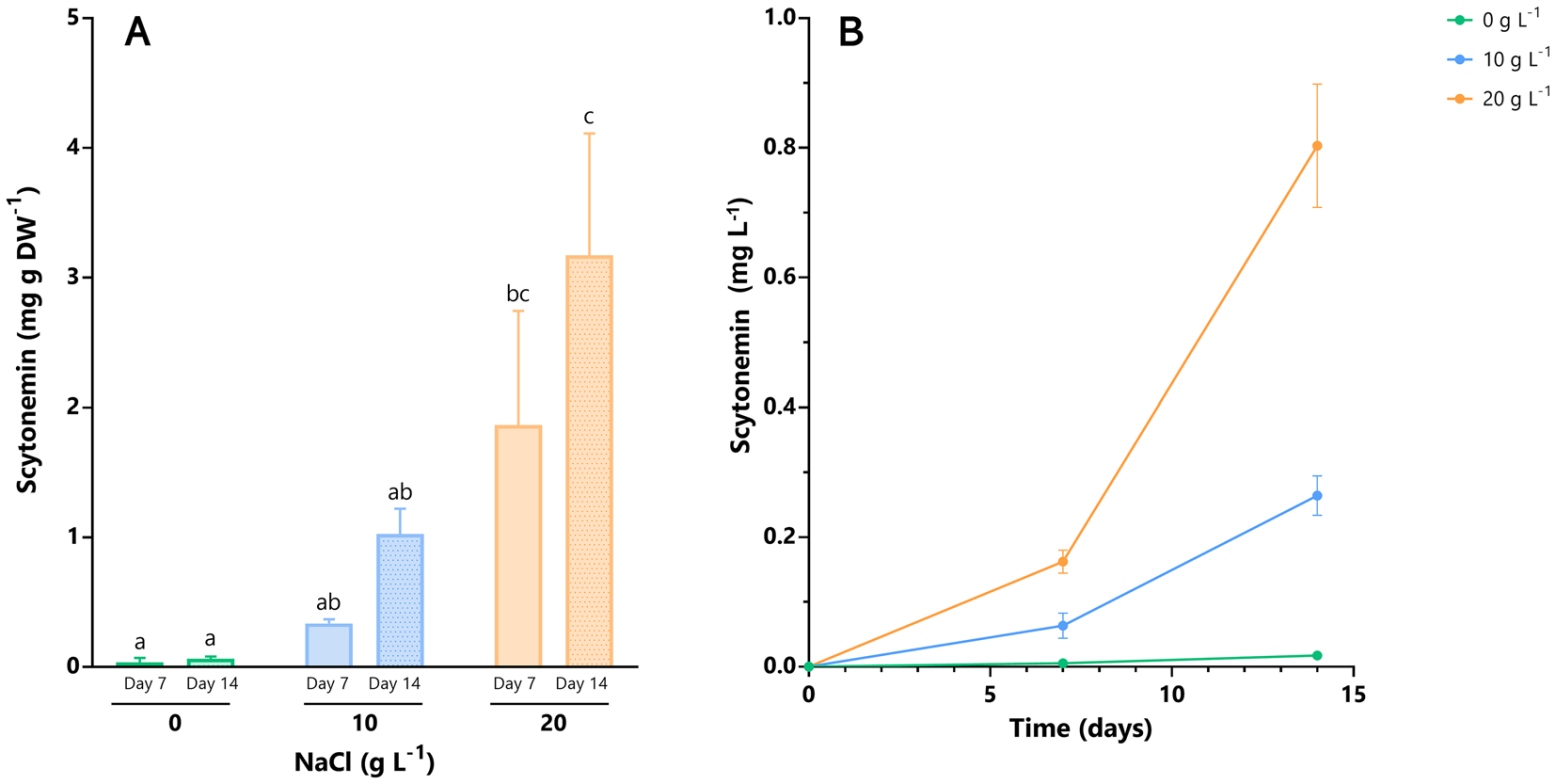
(**A**) Scytonemin content in *Chroococcidiopsis* sp. UAM571 grown in BG11 with 0 g L^−1^ (green), 10 g L^−1^ (blue) and 20 g L^−1^ (orange) NaCl on days 7 (plain bars) and 14 (dotted bars) of experiment. (**B**) Evolution of total scytonemin concentration in the culture of *Chroococcidiopsis* UAM571 in BG11 with 0 g L^−1^, 10 g L^−1^ and 20 g L^−1^ NaCl. The content is expressed in mg scytonemin g^−1^ of DW (**A**) and mg scytonemin L^−1^ culture (**B**). Error bars represent SD (n = 3). The same letter indicates the absence of significant differences (p > 0.05; ANOVA, Bonferroni).

The study of the scytonemin content of *Chroococcidiopsis* sp. UAM571 revealed that its value increased through time and higher NaCl concentrations (Fig. 1A, Table 2). Thus, the highest scytonemin content (3.17 mg g DW^−1^) was obtained when it was exposed to 20 g L^−1^ NaCl-supplemented BG11 for 14 days, which represented more than 0.3% of biomass DW. This scytonemin content value was significantly higher compared to the control culture, reaching a drastic 53-fold difference, and to its exposure to 10 g L^−1^ NaCl, reaching a threefold difference (< 0.05; ANOVA, Bonferroni). The exposure to 10 g L^−1^ NaCl supplemented media also showed an increase in scytonemin content, up to 17 times higher than the one obtained in control conditions (Table 2). However, no significant differences were found between scytonemin content values under this NaCl concentration and control conditions. On the other hand, even though a difference of threefold and almost twofold was observed when comparing the scytonemin content between exposure times, this factor resulted non-significant for any of the NaCl concentrations (p > 0.05; ANOVA, Bonferroni) (Table 2, Fig. 1A).

**Table 2:** *Chroococcidiopsis* sp. UAM571 scytonemin content and productivity (g DW L^−1^ day^−1^) grown on BG11 supplemented with different NaCl concentrations.

NaCl (g L^−1^)	Scytonemin content (mg scytonemin g DW^−1^)		
	Day 7	Day 14	Productivity (µg scytonemin L^−1^ day^−1^)
0	0.04 ± 0.03^a^	0.06 ± 0.02^a^	1.2 ± 0.3^a^
10	0.34 ± 0.03^ab^	1.03 ± 0.20^ab^	18.9 ± 2.2^b^
20	1.86 ± 0.88^bc^	3.17 ± 0.94^c^	57.4 ± 6.8^c^

Values are expressed as mean ± standard deviation (SD) (n = 3).

The same letter indicates no significant differences (p > 0.05; ANOVA, Bonferroni).

In contrast to scytonemin content results, the scytonemin concentration observed at both exposure times (7 and 14 days) of the highest NaCl concentration tested showed significant differences between them and with the rest of culture conditions studied (p < 0.05; ANOVA, Bonferroni) (Fig. 1B). The highest scytonemin concentration (0.80 mg L^−1^) was observed for 20 g L^−1^ NaCl-supplemented culture after 14 days.

When analysing the scytonemin productivity, it increased with NaCl concentration, reaching its maximum when the culture was grown under 20 g L^−1^ NaCl-supplemented BG11 (57.4 µg L^−1^ day^−1^) (Table 2) and 3 times higher than the one observed at 10 g L^−1^ NaCl. The statistical analysis revealed significant differences in scytonemin productivity between all NaCl concentrations tested (< 0.05; ANOVA, Bonferroni).

**Figure 2:**
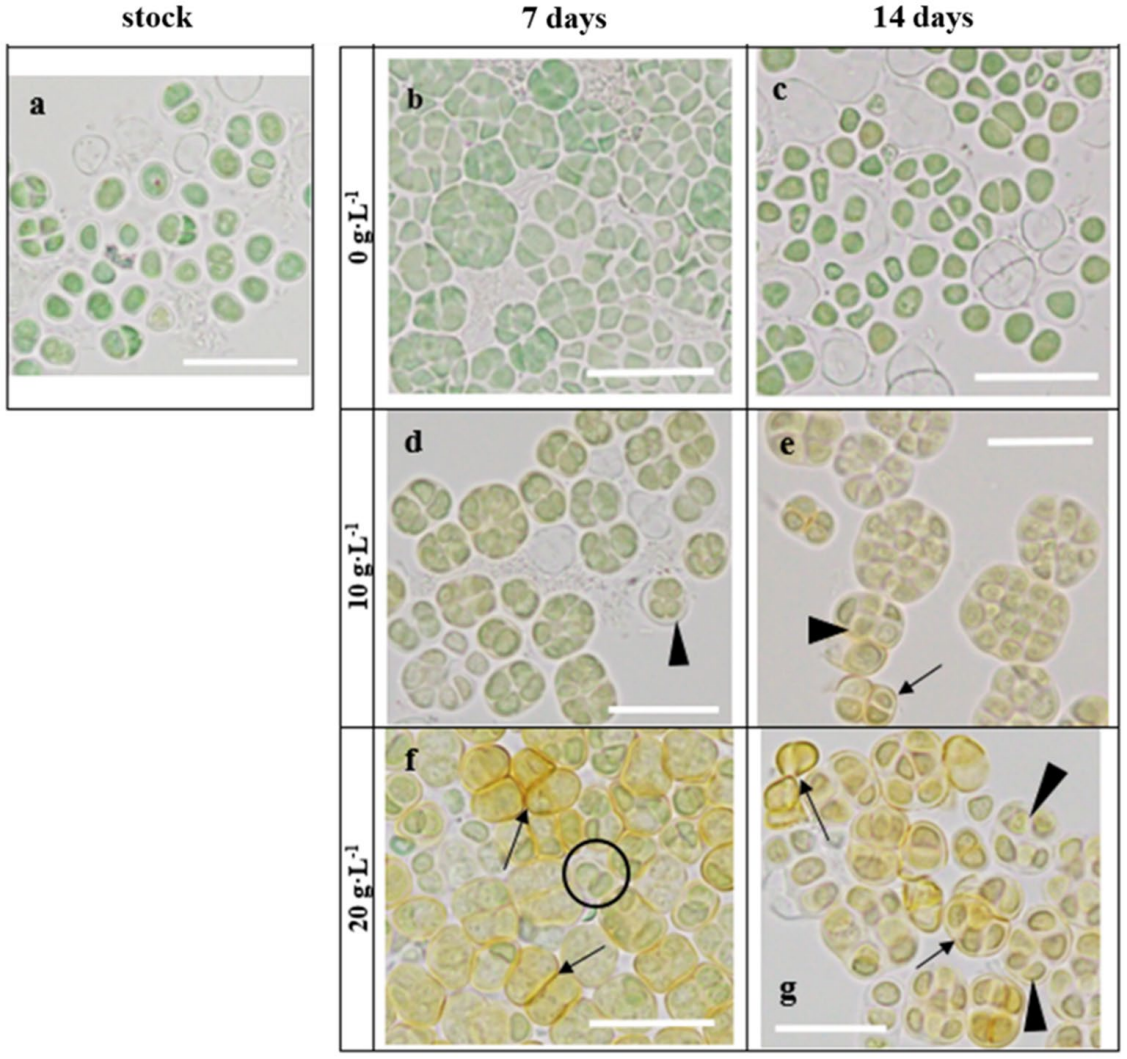
Bright field microscopy images of *Chroococcidiopsis* sp. UAM571 cells during scytonemin production experiment. (**a**) Stock culture; (**b,c**) cells exposed to control conditions (0 g L^−1^ NaCl) after 7 and 14 days; (**d,e**) cells exposed to 10 g L^−1^ NaCl supplemented BG11 culture medium after 7 and 14 days; (**f,g**) cells exposed to 20 g L^−1^ NaCl supplemented BG11 culture medium after 7 and 14 days. Scale bar indicates 20 µm. Black arrowheads point to EPSs, arrows point to scytonemin associated to EPSs capsules, black circle indicates green cells in 20 g L^−1^ NaCl supplemented BG11 culture.

The increasing scytonemin production with time and NaCl concentration was also observed in the microscopy images as an increasing intensity of brown-yellow color in the EPSs layer of the cells (Fig. 2). These changes in color were also accompanied by a higher EPSs layer thickness when cells were exposed to increasing NaCl concentrations (Fig. 2d–g). were scytonemin was accumulated (Fig. 2e–g). To confirm this apparent increase in EPS we performed an extraction and spectrophotometric determination in samples exposed to 20 g NaCl L^−1^ and 0 g NaCl L^−1^. The quantification supported microscopic findings since UAM571 showed an average EPS content of 35.4 mg EPSs g^−1^ DW under 20 g NaCl L^−1^ compared to a 1.4-fold reduced production without NaCl (25.2 mg EPSs g^−1^ DW on average).

### Phylogenetic study of *scyC* gene

**Figure 3:**
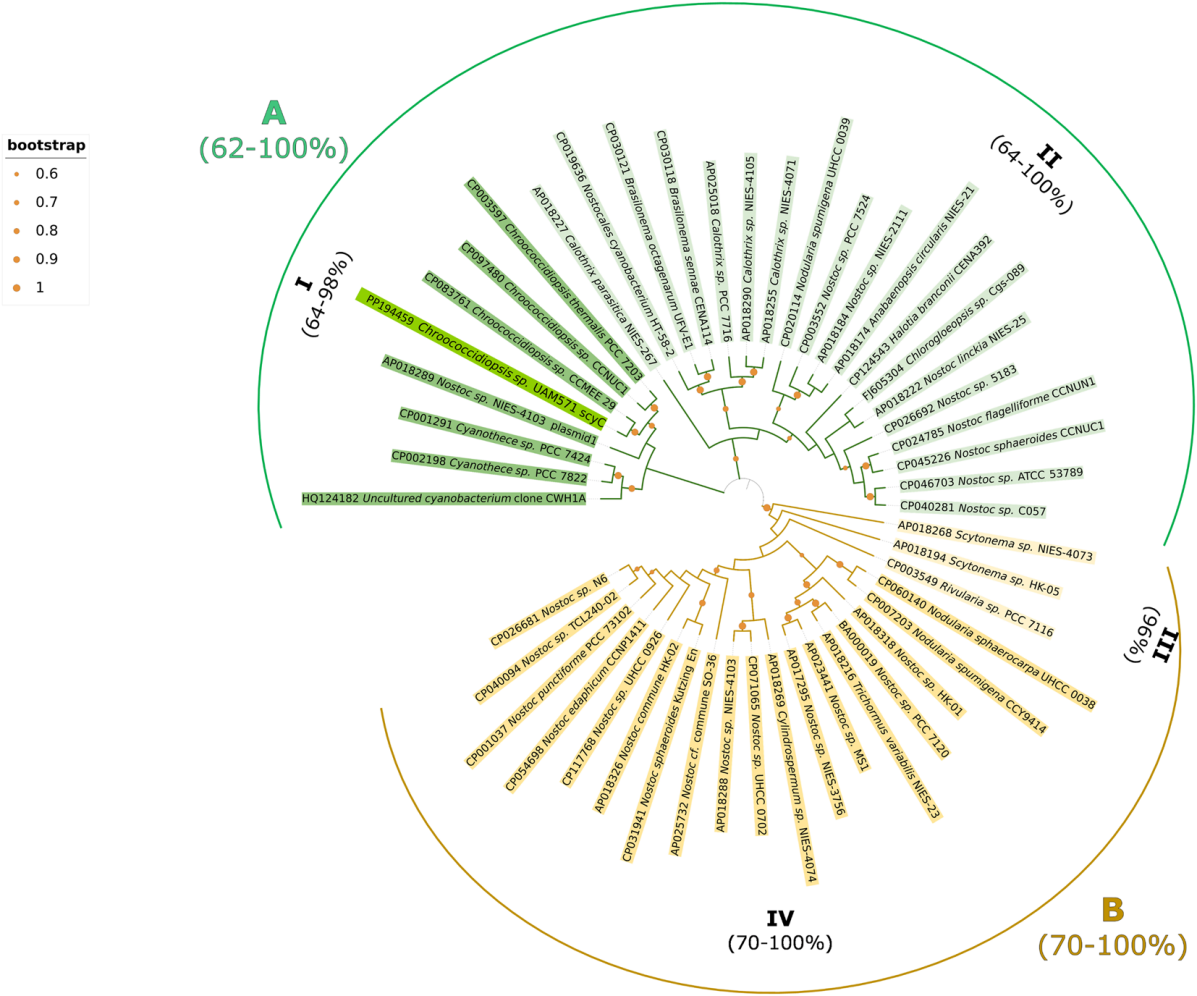
Phylogenetic tree according to the Maximum Likelihood principle based on the sequences of 48 cyanobacterial strains for the *scyC* gene. Sequences conforming cluster A (containing subclusters I and II) are highlighted in green. Sequences conforming cluster B (containing subclusters III and IV) are highlighted in yellow. The sequence of this study is highlighted in bright green. Sequence similarity range of each cluster and subcluster is indicated in brackets. Bootstrap values above 60% are indicated by orange sized dots at each branch.

The comparison of *scy C* gene sequence from *Chroococcidiopsis* sp. UAM571 (515 bp) using BLAST tool from NCBI showed the highest similarity with *Chroococcidiopsis* sp. CCMEE 029 strain (CP083761) with a percent of identity of 90.70%. This result was consistent with the phylogenetic study of this gene (Fig. 3) where UAM571 *scyC* sequence was grouped with the one belonging to CCMEE 029 strain and others from the same genus, *Chroococcidiopsis*. The subcluster I (cluster A) where the *scyC* sequence of this study was located showed an intra-group similarity range of 64–98%. Cluster A is mostly composed of cyanobacteria isolated from terrestrial environments (*Anabaenopsis circularis* NIES-21, *Nostoc linckia* NIES-25, *Cyanothece* sp. PCC7822, *Chroococcidiopsis* sp. CCMEE29), while cyanobacteria conforming Cluster B belong originally to both aquatic *Rivularia* sp. PCC 7116, *Nodularia spumigena* CCYA9414 and *Nostoc edaphicum* CCNP1411) and terrestrial environments (*Cylindrospermum* sp*.* NIES-4074, *Nostoc* sp. NIES-3756).

## Discussion

Cyanobacteria are known to produce a large variety of bioactive compounds and those inhabiting such an extreme habitat as the Atacama Desert, especially in terms of solar radiation, are expected to produce compounds to deal with that extreme UVR as scytonemin, a UV screening compound synthesized exclusively by certain members of this phylum. However, the challenge of isolating, characterizing, and growing these organisms under laboratory conditions results in scarce information about the genes involved in the biosynthetic pathway of this compound, and the influence of different abiotic factors on their production^10–12^. Thus, our study aims to evaluate growth and scytonemin production capacity of cyanobacterial strains from the Atacama Desert under different NaCl concentrations, and to find their position within the evolutionary context based on the *scyC* gene.

Eight endolithic cyanobacterial strains belonging to the genera *Chroococcidiopsis, Gloeocapsa* and *Gloeocapsopsis* previously isolated from gypcrete and calcite from the Atacama Desert^25,26^ were used in this study. After a preliminary screening based on their growth rate in control conditions and under different NaCl concentrations, *Chroococcidiopsis* sp. UAM571 was selected to specifically study its growth and scytonemin production capacity when exposed to NaCl. Growth rates of endolithic cyanobacteria at their original habitat remain undetermined, although Davila et al.^27^ described that, in halite, these microbial communities count on conditions for photosynthesis only 10 days per year. Despite the expected low growth rate for *Chroococcidiopsis* sp. UAM571 in its original habitat, the observed growth rates in this study without NaCl (0.24 day^−1^) were similar to other strains from the same genus under the same conditions, 0.11–0.4 day^−1^^10,28^. However, this growth rate showed a 1.5 to tenfold difference with others from cyanobacteria isolated from aquatic environments such as *Microcystis aeruginosa* (0.29–0.62 day^−1^), *Cylindrospermopsis raciborskii* (0.67 day^−1^) and *Anabaena flos-aquae* (1.41 day^−1^)^29,30^.

Salinity is a crucial parameter in the screening for commercial production of cyanobacteria and microalgae since it can be utilized to cope with cross-contamination of the culture system by non-target microalgae/cyanobacteria and other microorganisms like bacteria, viruses, fungi and zooplankton (e.g., ciliates, copepods, rotifers)^31^. Despite salinity has been described as a limiting factor for growth in cyanobacteria isolated from non-hypersaline environments as the freshwater *Microcystis aeruginosa,* or *Cylindrospermopsis* sp. and *Anabaena* sp. from brackish environments, whose survival rates decreased at 6–15 g L^−1^ NaCl^32^, *Chroococcidiopsis* sp. UAM571 showed a broad tolerance to NaCl, up to 20 g L^−1^. Moreover, the observed growth rate at 20 g L^−1^ was only 1.7 times lower when compared to the control, similar to the one observed in *Chroococcidiopsis* sp. M88-VD-G for the same conditions (1.6 times)^10^, although its growth rate when exposed to NaCl was not as high as the one from the endolithic *Leptolyngbya* sp. ISTCY10 (0.25 day^−1^ at 25 g L^−1^ NaCl)^33^. Besides the effect in growth, microscopic observations and spectrophotometric quantification indicated higher production of EPSs in UAM571 related to NaCl exposure, which is coherent with previous findings in other cyanobacteria^34,35^. That higher EPSs production in *Chroococcidiopsis* sp. UAM571 exposed to NaCl presents another advantage for its commercial production since the EPSs make easier the self-flocculation and, therefore, the concentration of biomass in a smaller volume, decreasing the expenses derived from biomass recovery.

The influence of abiotic factors in scytonemin production is often studied by combining them with UVR exposure^6,10,11,25^, while only a few studies worked on osmotic stress as a single factor to induce scytonemin synthesis^10,12^. In this context, this study improves the knowledge in NaCl as a single stressor for scytonemin production, which, in addition to allowing mass cultivation as previously mentioned, will drop the production costs. The increasing NaCl concentration effect on scytonemin production by UAM571 was previously observed in M88-VD-G strain from the same genus^10^ and *Lyngbya aestuarii*^12^, that reached maximum scytonemin concentrations at 20 and 56 g L^−1^ NaCl respectively. The high tolerance and productivity observed in the latter cyanobacterial strain compared to *Chroococcidiopsis* sp. UAM571 could be explained by the isolation source of this *Lyngbya aestuarii* strain, a salty lagoon, thus this organism probably counts on adaptation strategies to grow and produce scytonemin exposed to NaCl. Considering the positive effect in scytonemin production by NaCl exposure, leading to a 53-fold increase in *Chroococcidiopsis* (this study^10^), it could be additionally multiplied by a factor of 10 when combined with UVR in this genus^10^. This points out to the combination of both factors (NaCl and UVR) as an interesting option to test in future studies in UAM571. However, any future experimental approach targeting biotechnological purposes should include detailed techno-economic analyses to evaluate whether the increased production with UVR counteracts the potentially higher costs of energy and specific materials (UVR light sources and transmitting materials for cyanobacterial growth) when simulating indoor large-scale cultivations.

The overall study of biomass and scytonemin productivity related to DW on *Chroococcidiopsis* sp. UAM571 suggests that, as a result of the increasing NaCl concentration, the relative quantity of EPS per cell would be higher^35^ providing a larger space for scytonemin to be deposited^36^. Besides this indirect effect of NaCl on scytonemin content via EPSs-synthesis triggering, it remains to be determined whether NaCl has a direct effect on scytonemin biosynthesis itself. One possibility may be to perform transcriptomics studies (e.g., with qPCR or RNA-Seq) selecting the most suitable *scy* genes once the scytonemin synthesis cluster is fully described in the genus *Chroococcidiopsis*.

Considering the growth and scytonemin production capacity of *Chroococcidiopsis* sp. UAM571 and the increasing biotechnological interest of scytonemin^7,18,37^ not yet commercially available, it seems crucial to estimate its potential mass productivity. With this purpose, we performed a simplified simulation of biomass (g DW m^−2^ day^−1^) and scytonemin productivity (mg m^−2^ day^−1^) based on our experimental data extrapolated to standard mass culturing devices including both a single system and two different multi-system arrangements (Table S2). Briefly, the single system would consist in a bubble column of 400 L and 0.3 m^2^^38^, while each multi-system column arrangement would propose a different spatial distribution of those systems on an extension of 1 hm^2^^39,40^. Therefore, single system growth of *Chroococcidiopsis* sp. UAM571 would produce a total biomass of up to 22 g DW m^−2^ day^−1^, which is similar to the biomass productivity found in commercially used cyanobacteria as *Arthrospira* spp. (8.2–27 g DW m^−2^ day^−1^) (rev.^41^) *Anabaena* sp. (9.4–23.5 g DW m^−2^ day^−1^)^42^. Regarding the pigments’ productivity, the highest scytonemin productivity would be 0.08 g·scytonemin m^−2^·day^−1^, about threefold lower than other cyanobacterial pigments with biotechnological interest as phycoerythrin and phycocyanin (2.6–2.8 g m^−2^ day^−1^)^38^ yet this might be counteracted by the higher potential market value of scytonemin compared to phycobiliproteins^43^. When simulating the multi-system arrangements displayed over 1 hm^2^, a biomass of 37–55 kg and up to 200 g of scytonemin could be produced by *Chroococcidiopsis* sp. UAM571 per day, even considering that productivity per m^2^ is reduced between 4 and 6 times depending on the arrangement (Table S2). In light of these promising estimates, future studies with UAM571 strain from Atacama may focus on performing indoor and outdoor cultivations to validate the actual profitability of mass producing scytonemin with this platform.

The biosynthetic pathway of scytonemin has not been studied deeply and annotated sequences of the genes involved in its production in NCBI GenBank are still scarce. However, the presence of the *scyC* gene has been published for at least 3 cyanobacterial genera, among them, *Nostoc*^44–46^, *Chlorogloeopsis*^47^ and *Nodularia*^48,49^ and could be found in whole-genome sequences of cyanobacterial strains belonging to other genera such as *Chroococcidiopsis*, *Cyanothece*, *Anabaena, Calothrix* and *Scytonema.* The use of specific primers for *scyC* amplification in the chosen cyanobacterial strain allowed to obtain a 515 bp sequence. The low sequence identity in 3 clusters of the phylogenetic tree (≤ 70%) (Fig. 3) point to *scyC* as a poorly conserved gene.Within the genus *Chrooccocidiopsis*, UAM571 is the only Atacama strain being the other strains isolated from Negev Desert (Israel) and from soils in Germany and China (Fig. 3), according to the information available for those sequences in the NCBI Genbank. It remains to be solved. Obtaining further *scyC* sequences from endolithic Atacama strains would help top disentangle whether they form a separate subgroup given the isolation and extreme characteristics of this Desert. Altogether thephylogenetic trends suggested by Fig. 3 highlight the need to further study the genes involved in scytonemin biosynthesis in different genera to better understand not just the evolutionary history of scytonemin synthesis but also the regulation and the influence of abiotic factors on its production.

In summary, according to the results achieved and the estimates, the production of scytonemin by the Atacama Desert endolithic cyanobacteria *Chroococcidiopsis* sp. UAM571 would be optimal at 20 g L^−1^ NaCl since it is between 3 and 5 times higher than when exposed to 10 g L^−1^ NaCl, while presenting a moderate biomass productivity. The obtained values of biomass and scytonemin productivity in this work allow its consideration for mass cultivation with biotechnological purposes following outdoor open cultivation approaches. This study also provides new prospects in phylogenomics of scytonemin biosynthetic pathway in the genus *Chroococcidiopsis*. Future studies elucidating its *scy* cluster infull might allow transcriptomics determination (e.g., via qPCR or RNA-Seq) of salinity effects, alone or in combination with UV exposure, on the production of this biotechnologically and ecologically relevant pigment while also providing new gene alternatives for its heterologous production in *E. coli*^18^.

## Methods

### Organisms and culture conditions

Growth experiments were conducted in 8 endolithic cyanobacterial strains isolated from samples taken in the Atacama Desert (Northern Chile) in 2015^25,26^ belonging to 3 different genera (*Chroococcidiopsis*, *Gloeocapsa* and *Gloeocapsopsis*), different microhabitats (cryptoendolithic, chasmoendolithic and hypoendolithic) and substrates (gypcrete and calcite) (Table 3). Their identification at genus level and phylogenetic relationships based on 16S rRNA can be found in previous publications^26^ and the sequences are available in the National Center for Biotechnology Information GenBank under accession numbers MW544037–MW544043. All cyanobacterial strains are maintained in BG11 liquid culture media^50^, 25 °C and 7 μmol photons·m^−2^·s^−1^ as part of the Universidad Autónoma de Madrid culture collection.

**Table 3:** Endolithic cyanobacterial strains used in the study.

Strain	Taxonomical assignment	Lithic substrate	Microhabitat	Location	References
UAM569	*Chroococcidiopsis*	Gypcrete	Chasmoendolithic	Monturaqui	^26^
UAM570	*Chroococcidiopsis*	Gypcrete	Cryptoendolithic	Monturaqui	^26^
UAM571	*Chroococcidiopsis*	Gypcrete	Hypoendolithic	Monturaqui	^26^
UAM572	*Gloeocapsa*	Gypcrete	Hypoendolithic	Monturaqui	^26^
UAM573	*Gloeocapsopsis*	Gypcrete	Hypoendolithic	Monturaqui	^26^
UAM579	*Chroococcidiopsis*	Gypcrete	Hypoendolithic	Cordón de Lila	^26^
UAM580	*Chroococcidiopsis*	Gypcrete	Hypoendolithic	Cordón de Lila	^26^
UAM586	*Chroococcidiopsis*	Calcite	Chasmoendolithic	Yungay	^25^

### Growth studies for strain selection

Ten-day growth curves were performed for the 8 study strains in 50 mL BG11 culture medium, under orbital shaking (135 rpm), at a temperature of 25 °C and constant light of 38.9 μmol photons m^−2^ s^−1^. An optical density at 750nm (OD_750nm_) of 0.1 was used as a starting point for all cultures performing biological triplicates for each strain. Growth was determined by measuring OD_750nm_ with a UV–Vis spectrophotometer (Hitachi U-2000 Spectrophotometer) on days 3, 7 and 10 of the experiment.

### Experiment on *Chroococcidiopsis* sp. UAM571

#### Effect of NaCl on growth

Fourteen-day growth curves were performed *Chroococcidiopsis* sp. UAM571 in 150 mL BG11 culture medium with 0, 10 and 20 g L^−1^ NaCl concentrations, performing biological triplicates. Conditions of starting point OD_750nm_, light, temperature and agitation were maintained as indicated in “Growth studies for strain selection” section.

#### Scytonemin content determination

Scytonemin content was analysed for each culture condition at days 7 and 14 of the experiment. The extraction and quantification were conducted as in Ref.^25^, with minor modifications. Briefly, cyanobacterial biomass of 5 mL of each experimental condition and replicate was collected through filtration on 0.2 µm pore-size polycarbonate filters (Cyclopore TM, Whatman). Filters were then suspended in 1:1 (v/v) methanol: ethyl acetate, agitated through vortex for 1 min and subjected to sonication bath for 10 min. Then the filters were removed, and the extracts incubated overnight at 4 °C in darkness to facilitate the extraction of scytonemin^6^. Finally, the extracts were filtered through 0.2 μm pore-sized sterilized syringe-driven filter (Symta, Madrid, Spain).

Scytonemin content was determined by measuring the scytonemin absorbance maximum at 384 nm, pooled carotenoids (490 nm), and that of chlorophyll *a* at (663 nm) and following previous equations from Ref.^9^. Extinction coefficient (ε) for scytonemin was 112.6 L g^−1^ cm^−1^^51^. Absorbance values were corrected for residual scatter by subtracting the absorbance at 750 nm. All absorbance measurements were made on Genesys 150 UV–Visible Spectrophotometer (Thermo scientific). All scytonemin measurements were normalized to dry weight (DW) obtained as described in Ref.^52^.

Finally, light microscopy observations of the cells were also performed to follow the production of scytonemin via their change in colour over time (from blue-green to yellow–brown as more scytonemin accumulates) using a OLYMPUS BX63 microscope with a equipped with a Leica DC 300 F digital camera (Leica Microsystems, Germany). All images were obtained with OLYMPUS cellSens Dimension software.

#### Exopolysaccharides (EPSs) quantification

Exopolysaccharides were quantified in cultures exposed to 0 and 20 g NaCl L^−1^ for 14 days. 10-mL culture aliquots of each condition and replicate were centrifuged (10,000×*g*; 30 min) and the biomass pellet stored at − 20 °C until processed. Extraction and quantification of EPSs followed the methods by Singh et al.^53^ including spectrophotometric determination^54^. The concentration of EPS (mg L^−1^) was standardized to biomass (dry weight) and expressed as content in mg EPS g^−1^ DW to allow comparisons between conditions.

### DNA extraction and, *scyC* gene PCR, sequencing, and phylogenetic analysis

Genomic DNA from the selected cyanobacterial strain was isolated with UltraClean Microbial DNA Isolation Kit (MO BIO Laboratories) following the manufacturer instructions. To increase cell lysis, 3 cycles of freeze-defreeze were performed using liquid nitrogen and a 60 °C thermal bath. PCR of the *scyC* gene from the scytonemin cluster was performed by using PfuTurbo Cx Hotstart DNA Polymerase (Agilent) and the primers specifically designed in this study scyChF (GTCTACTTCCATTGG) and scyChR (CCGCTTGATATTCAT) with a target region of 570 bp, applying 1 cycle of denaturalization at 95 °C for 1 min, followed by 35 cycles of 95 °C for 30 s, annealing step at 43 °C for 1 min and elongation at 72 °C for 1 min, finalizing with an elongation step at 72 °C for 5 min. Sequencing of PCR product was performed by using the kit Big DyeTM Terminator v3.1 Cycle Sequencing (Applied Biosystems) in a ABI Prism 3730 Genetic Analyzer (Applied Biosystems) at the Spanish National Center of Oncology Research (CNIO, Madrid).

For the phylogenetic analysis, obtained sequence of the *scyC* gene was aligned with sequences obtained from the NCBI GenBank (NCBI MegaBlast tool http://blast.ncbi.nlm.nih.gov/Blast.cgi, accessed 18.07.23) using the Clustal W 1.4 software^55^. The final alignment length was 515 bp. A phylogenetic tree was constructed in MEGA11 software 11.0.13 version^56^ using the Maximum Likelihood (ML) method. The best-fitting evolutionary model, chosen following the BIC (Bayesian Inference Criterion), was Tamura-3 model with gamma distribution^57^. 1000 bootstrap replicates were performed for the tree.

### Statistical analysis

Statistical comparisons were performed using one- and two-factor ANOVA tests and the Bonferroni post-hoc test. A significance level of p = 0.05 was used in all cases. All statistical analyses were carried out using GraphPad Prism 9.1.0.221.

### Supplementary Information

Supplementary Information.

## Data Availability

The DNA sequence of *Chroococcidiopsis* sp. UAM571 *scyC* gene generated and analysed during the current study is available in the GenBank repository, under the Accession Number PP194459.
