# Supplementary material for: Effect of salinity on scytonemin yield in endolithic cyanobacteria from the Atacama Desert
Source: Sci Rep. 2024 Apr 28;14:9731. doi: 10.1038/s41598-024-60499-4 (PMC11056366; doi:10.1038/s41598-024-60499-4)
Supplement: Supplementary file 1 — Supplementary Information. [file 41598_2024_60499_MOESM1_ESM.docx]

**Supplementary material**

**Table S1. Growth rates (d^-1^) *Chroococcidiopsis* sp. UAM571 and *Gloeocapsa* sp. UAM572 on BG11 supplemented with different NaCl concentrations.** Values are expressed as mean ± standard deviation (SD) (n=3). * Indicates significant differences between NaCl concentrations on each cyanobacterial strain (p<0.05; ANOVA, Bonferroni).

**Growth rate (d^-1^)**

**NaCl (g L^-1^)** ***Chroococcidiopsis* sp. UAM571 *Gloeocapsa* sp. UAM572**

0 0.24 ± 0.01 0.22 ± 0.01

5 0.24 ± 0.01 0.19 ± 0.01

10 0.22 ± 0.01 0.19 ± 0.01

20 0.14 ± 0.01 0.12 ± 0.01

30 -0.06 ± 0.01* -0.03 ± 0.01*

**Figure S1**. Growth curves of the 8 endolithic cyanobacterial strains in BG11 culture medium expressed by OD_750nm_. Error bars represent SD (n=3).


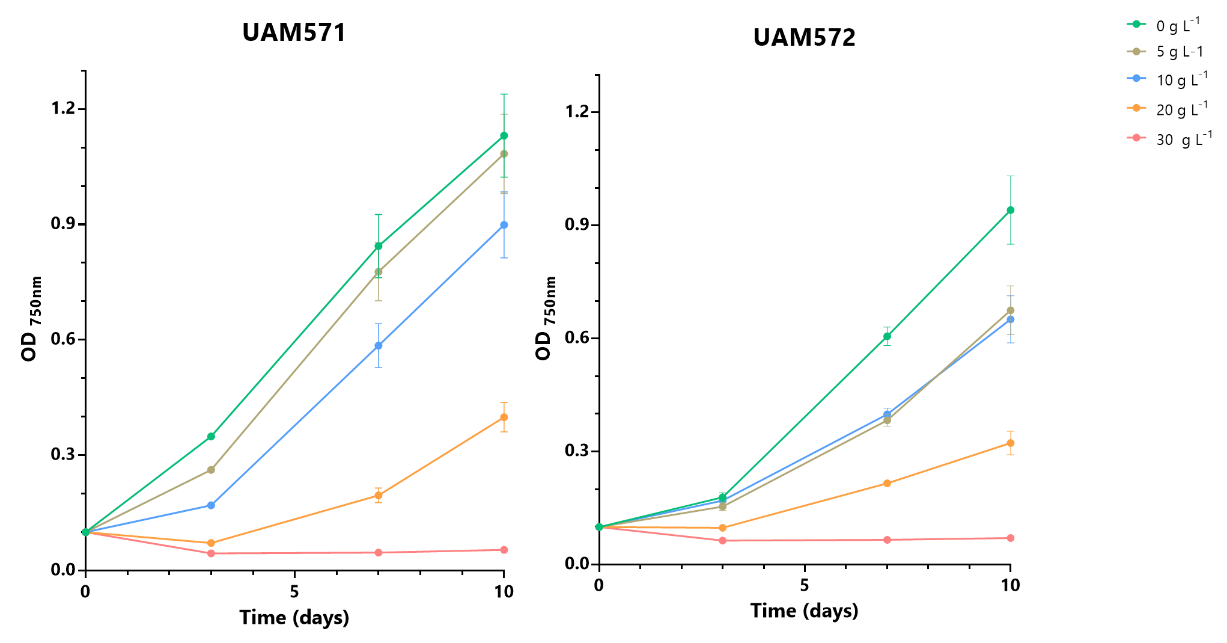


**Figure S2.** Growth curves of *Chroococcidiopsis* sp. UAM571 (a) and *Gloeococapsa* sp. UAM572 (b) on BG11 with different NaCl concentrations. Error bars represent SD (n=3).

**Figure S3.** Growth curves of *Chroococcidiopsis* sp. UAM571 on BG11 with different NaCl concentrations. Error bars represent SD (n=3).

**Table S2. Simulated biomass and scytonemin productivity in single and multi-system arrangements mass production for *Chroococcidiopsis* sp. UAM571.**Values are expressed as mean ± standard deviation (SD) (n=3). Simulated multi-system productivities calculated considering east–west-oriented rows with inter-system spacing avoiding shadowing between systems according to two arrangements of bubble tank farms proposed in the literature: ^A^Wall-to-wall and inter-row separations from Chini Zittelli et al. (2006), ^B^Wall-to-wall and inter-row separations proposed by Mirón et al. (1999).

**Biomass (g DW m^-2^ d^-1^)**  **Scytonemin (mg m^-2^ d^-1^)**

**NaCl (g·L^-1^) Single**  **Arrangement A Arrangement B Single Arrangement A Arrangement B**

0 22.8 ± 0.6 5.7 ± 0.2 3.8 ± 0.1 1.6 ± 0.4 0.4 ± 0.1 0.3 ± 0.1

10 22.0 ± 6.0 5.5 ± 1.5 3.7 ± 1.0 25.1 ± 2.9 6.3 ± 0.7 4.2 ± 0.5

20 21.9 ± 5.1 5.5 ± 1.3 3.7 ± 0.9 76.5 ± 9.1 19.1 ± 2.3 12.7 ± 1.5
